# Subcortical and brainstem hemiatrophy accompanied by iron deposition in a patient with hemiparkinsonism-hemiatrophy syndrome: a case report

**DOI:** 10.1186/s12883-021-02080-4

**Published:** 2021-02-03

**Authors:** Su Jin Chung

**Affiliations:** Department of Neurology, Myongji Hospital, Hanyang University College of Medicine, 55, Hwasu-ro 14beon-gil, Deogyang-gu, Goyang, 10475 South Korea

**Keywords:** Hemiparkinsonism, Hemiatrophy, Subcortex, Brainstem, Iron

## Abstract

**Background:**

There is no established pathogenesis of hemiparkinsonism-hemiatrophy syndrome (HPHA), and the varied clinical presentations have been reported in several case studies. To the best of our knowledge, the present report describes the first case of HPHA with unusual brain imaging findings.

**Case presentation:**

A 20-year-old man presented with a 6-month history of weakness and clumsiness in his right limbs. He showed right-sided parkinsonism with dystonic hand posture; however, body asymmetry was not noted. Brain imaging revealed hemiatrophy of the left hemisphere subcortical structures and brainstem, and iron deposition in the left globus pallidus and substantia nigra. In addition, dopamine transporter imaging demonstrated normal presynaptic dopaminergic function. The patient was treated with levodopa, which had little to no effect.

**Conclusions:**

This case demonstrates the unique imaging characteristics of HPHA associated with widespread brain hemiatrophy and iron deposition. Further studies are needed to elucidate the diagnostic criteria for this heterogeneous syndrome.

**Supplementary Information:**

The online version contains supplementary material available at 10.1186/s12883-021-02080-4.

## Background

Hemiparkinsonism-hemiatrophy syndrome (HPHA) is a rare neurological syndrome characterized by unilateral atrophy on one side of the body or brain with slowly progressive hemiparkinsonism [1]. This clinical syndrome differs from idiopathic Parkinson’s disease (PD) in that it usually occurs in young adults and shows diverse responses to levodopa. Accompanying dystonic movements are frequent, and some cases have exhibited pyramidal signs. Many described patients had perinatal cerebral insults, febrile infections, or head trauma in early childhood. However, the underlying pathogenesis of this condition is not fully understood [2]. There are no published pathologic data on HPHA. Here, we present a case of HPHA with specific brain imaging findings that have never been reported.

## Case presentation

**Fig. 1:**
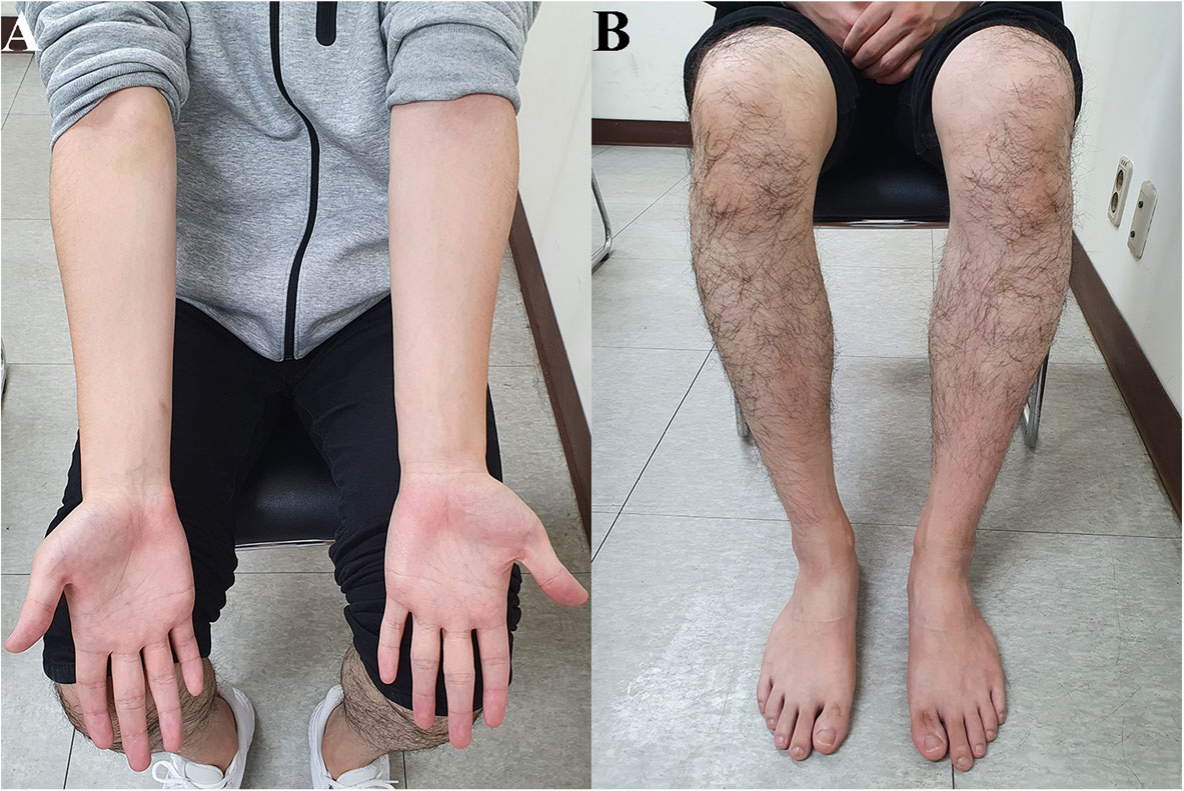
Photographs of the case. The patient shows symmetric body mass in the upper (**a**) and lower extremities (**b**)

**Fig. 2:**
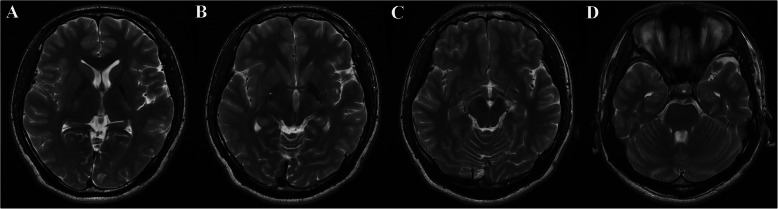
T2-weighted images of the patient. Subcortical and brainstem structures show atrophic changes in the left basal ganglia (**a**), thalamus (**b**), midbrain (**c**), and pons (**d**). Furthermore, dense hypointensity was seen in the left globus pallidus (**a, b**), with light hypointensity in the left substantia nigra (**c**)

**Fig. 3:**
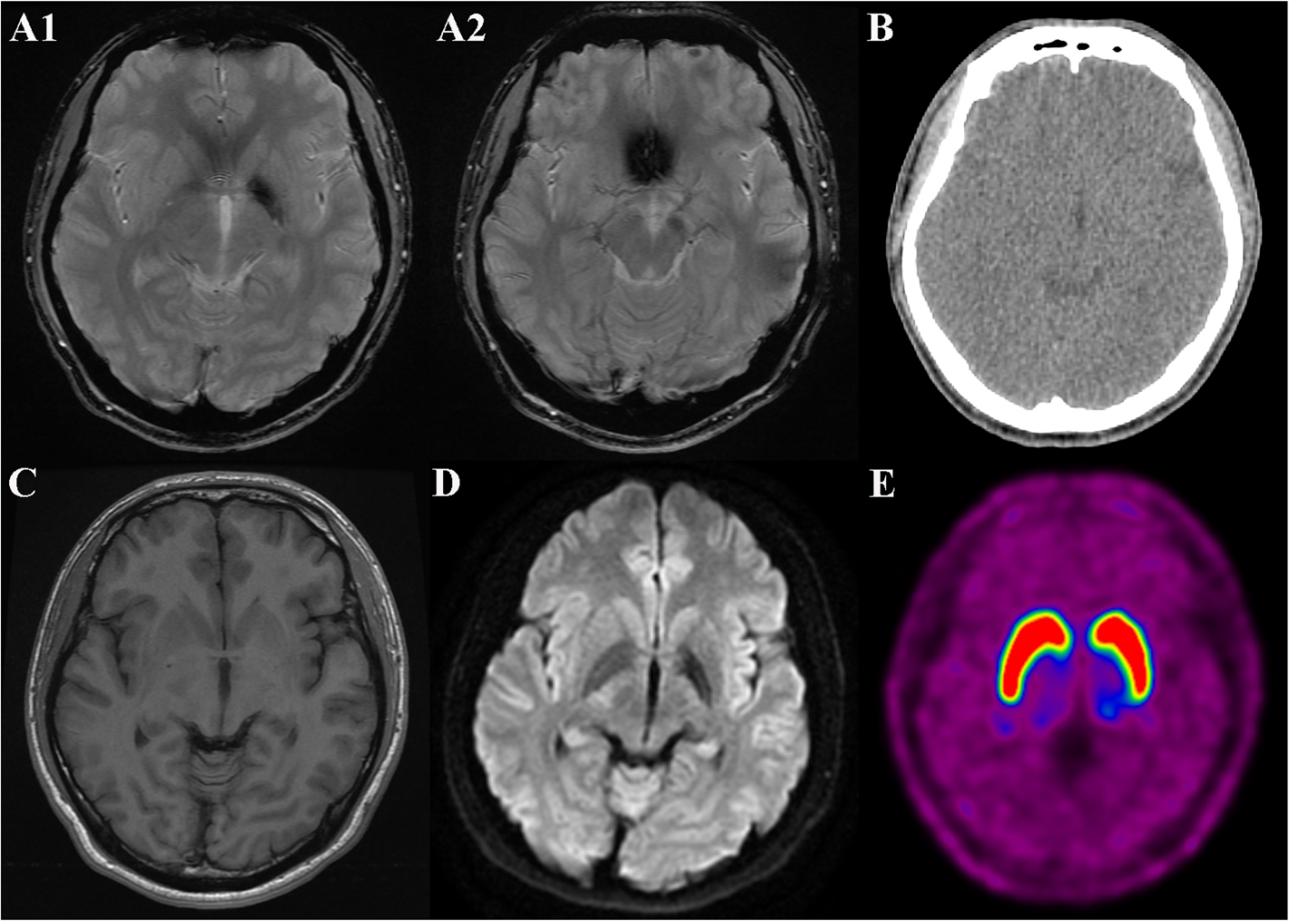
Neuroimaging of the patient. T2*-weighted gradient-echo sequences reveal the presence of hypointensities within the left globus pallidus (**a1**) and left substantia nigra (**a2**). The left globus pallidus shows no signal change on computed tomography scan (**b**) or T1-weighted image (**c**), while a low signal abnormality is seen on diffusion-weighted imaging (**d**). The [^18^F] N-(3-fluoropropyl)-2β-carbon ethoxy-3β-(4-iodophenyl) nortropane positron emission tomography scan shows no pathological striatal alterations (**e**)

A 20-year-old man visited our hospital due to a 6-month history of weakness and clumsiness in his right extremities. His past history was negative for perinatal problems. There was no relevant family history for neurodegenerative diseases. He did not exhibit any learning difficulties in university courses and denied a history of psychiatric illness. On physical examination, body asymmetry was not visually apparent across the whole body, and differences in opposite limb circumferences did not exceed 2 cm (10 cm below the elbow, right: 24 cm, left: 25 cm; 10 cm below the knee, right: 35 cm, left: 35 cm) (Fig. 1). Dystonic postures of the proximal interphalangeal joints of the right hand were found when he outstretched both arms, with hyperextension of the thumb and flexion of the little finger. Neurological examination demonstrated subtle weakness of his right limbs with hyperreflexia and pathologic reflexes. Repetitive limb movements were slow with decremental responses in the right hand and foot. The right extremities showed mild rigidity without resting or postural tremor. His gait was characterized by decreased right arm swing, but his base and stride seemed normal (See Additional file 1). His Unified Parkinson’s Disease Rating Scale (UPDRS)-Part III motor score was 8/108. Laboratory results were all within normal limits, including serum copper, ceruloplasmin, iron, ferritin, manganese, and 24-h urine copper levels. Assessment of cognitive function revealed normal (30/30 points on the Mini-Mental State Examination and 30/30 points on the Korean Version of the Montreal Cognitive Assessment). Brain magnetic resonance imaging (MRI) revealed remarkable subcortical and brainstem hemiatrophy in the left basal ganglia, thalamus, midbrain, and pons (Fig. 2). In addition, hypointense signals were observed on T2-weighted images and T2*-weighted (T2*W) gradient-echo (GRE) sequences in the left globus pallidus and substantia nigra. These lesions were isointense on computed tomography scans and T1-weighted images, and hypointense on diffusion-weighted imaging sequences (Fig. 3a-d). These findings were compatible with iron accumulation rather than calcification or haemorrhage. On the contrary, [^18^F] N-(3-fluoropropyl)-2β-carbon ethoxy-3β-(4-iodophenyl) nortropane (^18^F-FP-CIT) positron emission tomography (PET) scans revealed normal levels of dopamine transporters (DATs) in the bilateral basal ganglia (Fig. 3e). The levodopa response was poor, but the patient expressed subjective improvement of his motor function. After a year of treatment, he still presented with unilateral parkinsonism in his right limbs without disease progression to the opposite side. His 1-year follow-up UPDRS-Part III motor score was 7/108.

## Discussion and conclusions

We describe a young patient with hemiparkinsonism and hemiatrophy of the brain that extended to the brainstem and was accompanied by iron deposition. He showed symptoms affecting both the extrapyramidal and pyramidal systems. The patient did not have any perinatal injuries or body mass asymmetry; however, other clinical features suggested the possibility of HPHA, which is characterized by early onset, slowly progressive, asymmetric hemiparkinsonism combined with contralateral cerebral hemiatrophy, dystonic postures, pyramidal signs, and poor response to levodopa. Above all, his unique brain structural imaging findings may be a new turning point in the diagnosis of this rare syndrome.

Although the term hemiatrophy has mostly implied a body asymmetry in previously reported cases, it can also mean a loss of volume in specific brain structures. Nevertheless, a number of previously published articles described a unilateral loss of body mass with or without brain hemiatrophy. However, the clinical definition of this syndrome indicates that body hemiatrophy is often present but not necessarily noted by the patient from early childhood [2]. Furthermore, Giladi et al. defined this syndrome as being characterized by marked asymmetry in parkinsonian signs, combined with atrophy of either the ipsilateral body side or contralateral cerebral hemisphere [1, 3]. In their study, they actually included a patient with hemiparkinsonism who had only contralateral brain hemiatrophy without ipsilateral body hemiatrophy [1]. In addition to our case, distinct brain asymmetry can lead to a diagnosis of HPHA, even if the patient does not have unilateral body side atrophy.

The MRI findings in patients with HPHA can be subdivided into the following categories: focal atrophy or diffuse cerebral hemiatrophy, single or multiple focal lesions in the basal ganglia, single or multiple focal lesions outside the basal ganglia, and normal imaging findings [2]. Brain structural damage is found in approximately 30% of patients with HPHA, which is typically characterized by cortical or subcortical hemiatrophy along with asymmetric ventricular enlargement [2]. Above all, the majority of reported cases had minimal ventricular asymmetry, with or without atrophy of the basal ganglia. Recently, one study reported a case of midbrain hemiatrophy and nigral rarefaction in a patient with HPHA [4]. To the best of our knowledge, this is the first report of a case of HPHA characterized by extensive brain hemiatrophy mainly affecting the subcortex and brainstem. Cerebral hemiatrophy can be caused by congenital factors, such as insults in utero, or acquired factors, such as birth trauma, infections, inflammation, vascular injuries, or neoplasms [5]. In our patient, the aetiology of cerebral hemiatrophy remained unclear. However, this case can broaden the perspective of MRI findings of HPHA.

Iron accumulation in the brain primarily occurs in areas associated with motor activity, including the globus pallidus, substantia nigra, red nucleus, and dentate nucleus [6]. In particular, the iron level in the globus pallidus is higher than that in any other brain regions, and the globus pallidus acts as an iron reservoir for the central nervous system [7]. Iron deposition in some brain regions usually increases with age, but excessive aggregation in particular brain regions is seen in a number of neurodegenerative diseases. It has been hypothesized that iron overload could increase neurotoxicity through the induction of oxidative reactions and cellular damage [8]. Whether iron concentrations cause direct neuronal injuries or are just secondary products of neurodegenerative processes, is yet to be determined. T2*W GRE sequences are generally used to assess iron deposition [9]. In general, iron deposition in the globus pallidus and substantia nigra should exclude the possibility of neurodegeneration with brain iron accumulation; however, the findings are usually bilateral in the majority of these disorders [10]. In the case of PD, many previous studies have suggested that a number of iron-related proteins and tau protein may have contributed to iron deposition [11]. A consistent result in PD is the accumulation of iron in the substantia nigra, while a consensus has not been reached regarding similar findings in other brain regions, such as the globus pallidus, red nucleus, and putamen [12]. Several studies in PD patients demonstrated associations of nigral and pallidal iron deposition with disease severity, including an aggravation of motor disability [13]. Furthermore, in the 6-hydroxydopamine model of PD, the degeneration of nigrostriatal dopaminergic neurons leads to iron overload, suggesting that iron accumulation in the substantia nigra may be secondary to neurodegeneration [14]. Compared with multiple explanations of the relationship between iron accumulation and PD, little is known about iron pathophysiology in HPHA. To our knowledge, there is only one case study of unilateral iron deposition in the putamen and caudate nucleus in a patient with HPHA [5]. In our case, iron deposition in the globus pallidus and substantia nigra may have been the result of atrophy of these structures and neurodegeneration, and the most vulnerable site is the globus pallidus, where the T2*W GRE hypointensity was denser than that in the substantia nigra. Contrary to a diverse pattern of iron accumulation in PD, iron overload was only seen within unilateral basal ganglia contralateral to the affected limb in these patients with HPHA.

Regarding dopaminergic therapy in patients with HPHA, the response is highly variable. In the literature, [^18^F]-fluorodopa PET scans have demonstrated decreased uptake in the unilateral or bilateral striatum in patients with HPHA [2]. Other PET studies assessing striatal dopamine D2 receptor binding capacity reported normal findings despite the poor levodopa responsiveness observed in these patients [15]. These inconsistent findings imply that HPHA is caused by a combination of pre- and postsynaptic nigrostriatal dopaminergic dysfunction. In our case, preservation of presynaptic dopaminergic neurons was demonstrated by the normal ^18^F-FP-CIT PET findings. Therefore, the minimal response to levodopa in our patient represents possible postsynaptic dopaminergic dysfunction. Otherwise, minor changes in presynaptic dopaminergic neurons were present but did not appear because depletion of striatal DAT activities was below the detection threshold. A previous study found that around 5.7–14.7% of patients diagnosed as early PD showed normal presynaptic dopamine imaging [16]. A different interpretation could be that the nigrostriatal dopaminergic pathway might not be substantially affected, such as in parkinsonism secondary to vascular, toxic, or inflammatory pathology involving mainly the basal ganglia. If so, our patient’s symptoms may have been triggered by disruption of other linkages between the basal ganglia and brainstem. Levodopa-induced fluctuations and dyskinesias were rarely observed in HPHA [2], which may indicate a possible lack of presynaptic nigrostriatal degeneration in this disease.

In conclusion, although there are a very small number of case studies on HPHA, a case of cerebral hemiatrophy that affects the subcortex and brainstem is a novel finding in this disease. Moreover, the influence of abnormal iron accumulation on the disease course of HPHA requires additional study. This case may expand the spectrum of imaging findings in HPHA, and further reports are warranted to establish the diagnostic criteria for this heterogeneous syndrome.

## Supplementary Information

**Additional file 1.** Video of the patient. An abnormal posture of the right hand is shown in this video when he was asked to keep his arms outstretched. He has mild bradykinesia affecting the right arm and leg. His gait is normal, apart from a reduced arm swing on the right. The Hoffman sign and ankle clonus are demonstrated in his right limbs.

## Data Availability

All data and material (Additional file 1: video) supporting our findings are contained within the manuscript.

## Abbreviations

HPHA: Hemiparkinsonism-hemiatrophy syndrome
PD: Parkinson’s disease
UPDRS: Unified Parkinson’s Disease Rating Scale
MRI: Magnetic resonance imaging
T2*W: T2*-weighted
GRE: Gradient-echo
^18^F-FP-CIT: [^18^F] N-(3-fluoropropyl)-2β-carbon ethoxy-3β-(4-iodophenyl) nortropane
PET: Positron emission tomography
DAT: Dopamine transporter
